# Clinical characteristics, management strategies and outcomes of patients with recurrent venous thromboembolism in the real world

**DOI:** 10.1038/s41598-022-26947-9

**Published:** 2022-12-23

**Authors:** Yugo Yamashita, Takeshi Morimoto, Kazushige Kadota, Toru Takase, Seiichi Hiramori, Kitae Kim, Maki Oi, Masaharu Akao, Yohei Kobayashi, Mamoru Toyofuku, Moriaki Inoko, Tomohisa Tada, Po-Min Chen, Koichiro Murata, Yoshiaki Tsuyuki, Yuji Nishimoto, Jiro Sakamoto, Kiyonori Togi, Hiroshi Mabuchi, Kensuke Takabayashi, Takao Kato, Koh Ono, Takeshi Kimura

**Affiliations:** 1 Department of Cardiovascular Medicine, Graduate School of Medicine, Kyoto University, 54 Shogoin Kawahara-Cho, Sakyo-Ku, Kyoto, 606-8507 Japan; 2 Department of Clinical Epidemiology, Hyogo College of Medicine, Nishinomiya, Japan; 3 Department of Cardiovascular Medicine, Kurashiki Central Hospital, Kurashiki, Japan; 4 Department of Cardiology, Kinki University Hospital, Osaka, Japan; 5 Department of Cardiology, Kokura Memorial Hospital, Kokura, Japan; 6 Department of Cardiovascular Medicine, Kobe City Medical Center General Hospital, Kobe, Japan; 7 Department of Cardiology, Japanese Red Cross Otsu Hospital, Otsu, Japan; 8 Department of Cardiology, National Hospital Organization Kyoto Medical Center, Kyoto, Japan; 9 Department of Cardiovascular Center, Osaka Red Cross Hospital, Osaka, Japan; 10 Department of Cardiology, Japanese Red Cross Wakayama Medical Center, Wakayama, Japan; 11 Cardiovascular Center, The Tazuke Kofukai Medical Research Institute, Kitano Hospital, Osaka, Japan; 12 Department of Cardiology, Shizuoka General Hospital, Shizuoka, Japan; 13 Department of Cardiology, Osaka Saiseikai Noe Hospital, Osaka, Japan; 14 Department of Cardiology, Shizuoka City Shizuoka Hospital, Shizuoka, Japan; 15 Division of Cardiology, Shimada Municipal Hospital, Shimada, Japan; 16 Department of Cardiology, Hyogo Prefectural Amagasaki General Medical Center, Amagasaki, Japan; 17 Department of Cardiology, Tenri Hospital, Tenri, Japan; 18 Division of Cardiology, Faculty of Medicine, Nara Hospital, Kinki University, Ikoma, Japan; 19 Department of Cardiology, Koto Memorial Hospital, Higashiomi, Japan; 20 Department of Cardiology, Hirakata Kohsai Hospital, Hirakata, Japan

**Keywords:** Cardiology, Diseases

## Abstract

There is a paucity of data on management strategies and clinical outcomes after recurrent venous thromboembolism (VTE). In a multicenter registry enrolling 3027 patients with acute symptomatic VTE, the current study population was divided into the following 3 groups: (1) First recurrent VTE during anticoagulation therapy (N = 110); (2) First recurrent VTE after discontinuation of anticoagulation therapy (N = 116); and (3) No recurrent VTE (N = 2801). Patients with first recurrent VTE during anticoagulation therapy more often had active cancer (45, 25 and 22%, P < 0.001). Among 110 patients with first recurrent VTE during anticoagulation therapy, 84 patients (76%) received warfarin at recurrent VTE with the median prothrombin time-international normalized ratio (PT-INR) value at recurrent VTE of 1.6, although patients with active cancer had a significantly higher median PT-INR value at recurrent VTE compared with those without active cancer (2.0 versus 1.4, P < 0.001). Within 90 days after recurrent VTE, 23 patients (20.9%) during anticoagulation therapy and 24 patients (20.7%) after discontinuation of anticoagulation therapy died. Active cancer was a major cause of recurrent VTE during anticoagulation therapy as a patient-related factor, while sub-optimal intensity of anticoagulation therapy was a major cause of recurrent VTE during anticoagulation therapy as a treatment-related factor, particularly in patients without active cancer.

## Introduction

Venous thromboembolism (VTE), including pulmonary embolism (PE) and deep vein thrombosis (DVT), is one of major health problems all over the world with a yearly incidence of around one case per 1000 person-years^1^. VTE has a long-term risk of recurrence, and the risk of recurrence after 5 years was reported to be 20–25% in unselected cohorts of patients with VTE^2^. Thus, the prevention of recurrence by anticoagulation therapy is important^3^. The risk of recurrence is highest in the acute phase after VTE diagnosis, and it decreases rapidly after initiation of anticoagulation therapy^4^. Nevertheless, a certain number of patients could develop recurrent VTE beyond the acute phase irrespective of the status of anticoagulation therapy. Moreover, recurrent VTE was reported to have a considerable impact on mortality (relative risk ratio: 3.24)^5^. However, evidence on the optimal management strategies after recurrent VTE is scarce^6^. Consequently, the current VTE guidelines provide limited recommendations based on low-quality evidence for the management strategies after recurrent VTE especially during anticoagulation therapy^7–10^. Data on the current real-world management strategies and outcomes after recurrent VTE would be important for understanding the current issues and unmet needs in patients with recurrent VTE like other cardiovascular diseases. Thus, we aimed to investigate the clinical characteristics, management strategies and outcomes of recurrent VTE, using a large practice-based large observational study in Japan.

## Methods

### Study population

The COMMAND VTE (COntemporary ManageMent AND outcomes in patients with Venous ThromboEmbolism) Registry is a physician-initiated, retrospective, multicenter cohort study in which consecutive patients with acute symptomatic VTE among 29 centers in Japan were included between January 2010 and August 2014. The design of the registry was previously reported in detail^11,12^. We searched the hospital databases for clinical diagnosis and imaging examinations, and enrolled consecutive patients who met the definition of acute symptomatic VTE diagnosed within 31 days from symptom onset during the study period^13^.

**Figure 1:**
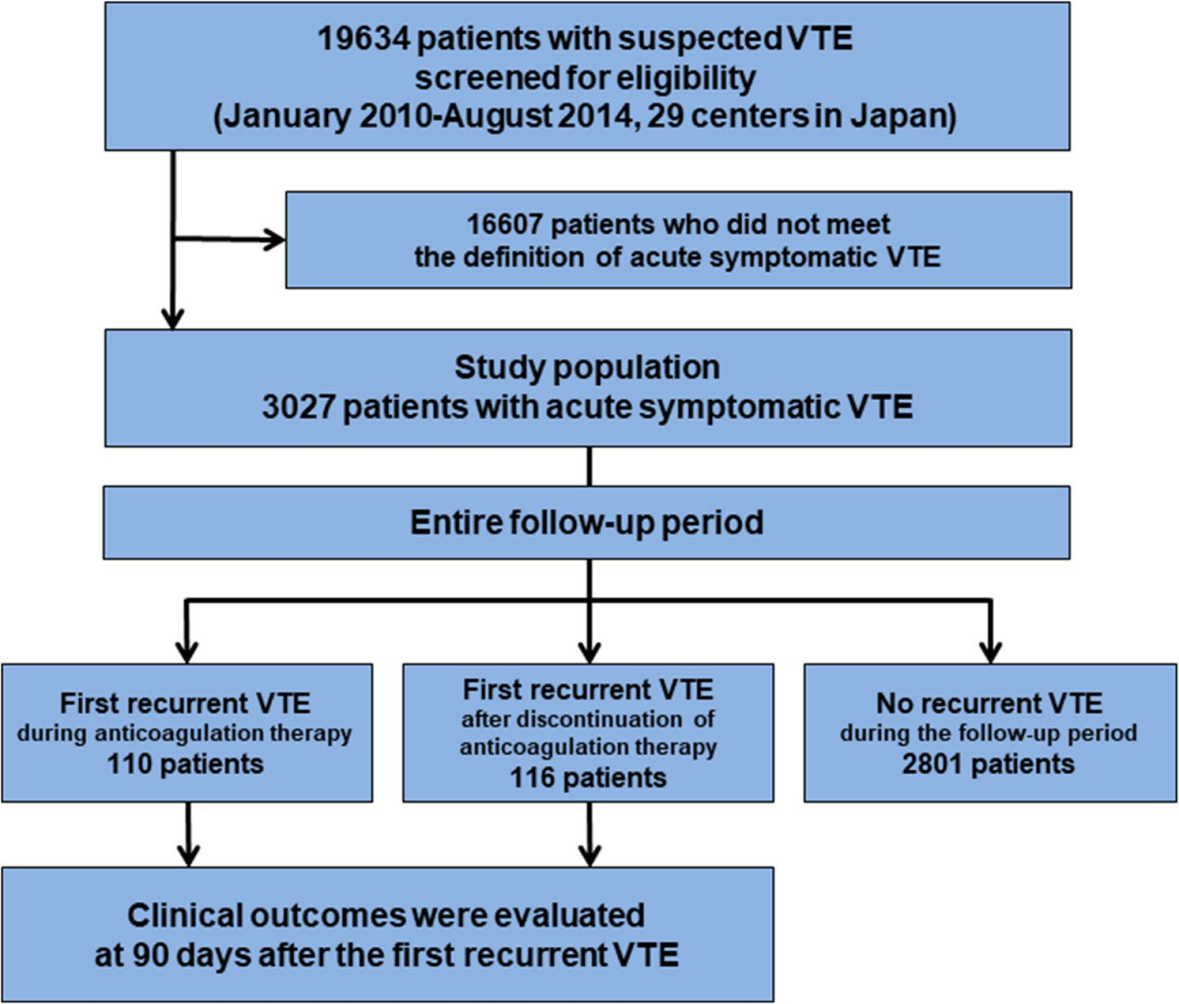
Study flowchart. VTE included PE and/or DVT. *DVT* deep vein thrombosis, *PE* pulmonary embolism, *VTE* venous thromboembolism.

We enrolled 3027 consecutive patients with acute symptomatic VTE after screening of the consecutive 19,634 patients with suspected VTE for eligibility through chart review by the physicians at each institution. According to the occurrence of recurrent VTE during the entire follow-up period and the status of anticoagulation therapy at the time of the recurrent VTE event, the current study population was divided into the following 3 groups; (1) First recurrent VTE during anticoagulation therapy; (2) First recurrent VTE after discontinuation of anticoagulation therapy; and (3) No recurrent VTE during the follow-up period (Fig. 1). Recurrent VTE was defined as PE and/or DVT with symptoms accompanied by confirmation of new thrombus or exacerbation of the thrombus by objective imaging examinations or autopsy^14^.

### Ethics approval and consent to participate

The ethics committee of primary institution; Kyoto University Hospital Ethics Committee approved the research protocol (Approval number R0493). The relevant review boards or ethics committees in all other 28 participating centers (Supplementary Appendix 1) also approved the research protocol. All procedures followed were in accordance with the Declaration of Helsinki. Written informed consent from each patient was waived because we used clinical information obtained in routine clinical practice and none of the patients refused to participate in the study when contacted for follow-up, which was approved by the ethics committee of the principal institution (Kyoto University Hospital Ethics Committee; the approval number R0493). This method is concordant with the guidelines for epidemiological studies issued by the Ministry of Health, Labor, and Welfare in Japan.

### Data collection and definitions for patient characteristics

Detailed methodology of data collection in the COMMAND VTE Registry was previously reported^11^. Just briefly, data were collected from the hospital charts or hospital databases according to the pre-specified definitions. The physicians at each institution were responsible for data entry into an electronic case report form with additional monitoring for the quality of data at the general office of the registry. Patients with active cancer were defined as those on treatment for cancer such as chemotherapy or radiotherapy, those scheduled to undergo cancer-surgery, those with metastasis to other organs, and/or those with terminal cancer (expected life expectancy of 6 months or less) at the time of the diagnosis. Data for prothrombin time-international normalized ratio (PT-INR) during follow-up in patients receiving warfarin were collected from the hospital charts of the centers where the index VTE was diagnosed. The presumed stable PT-INR values for warfarin users was evaluated as the first PT-INR between 1 and 6 months after VTE diagnosis. The detailed definitions of patient characteristics are described in Supplementary Appendix 2.

### Clinical follow-up and endpoints

Detailed methodology of clinical follow-up in the COMMAND VTE Registry was previously reported^11^. In the current study, we evaluated the clinical outcomes at 90 days after the first recurrent VTE events, and all the clinical outcomes were evaluated irrespective of the status of anticoagulation therapy after the first recurrent VTE.

The primary outcome measure in the current study was all-cause death at 90 days after the first recurrent VTE. The independent clinical event committee (Supplementary Appendix 3) unaware of the patient characteristics reviewed all the death events, and classified the causes of death as due to PE, due to bleeding events, due to cancers, due to cardiac causes, due to other non-cardiac causes, or due to unknown causes^15^. Death was judged to be due to PE (fatal PE) if it was confirmed by autopsy or if death followed a clinically severe PE after first recurrent PE events. Death was judged to be bleeding related if it followed an intracranial hemorrhage or a bleeding episode leading to hemodynamic deterioration. Death in patients with the end-stage cancer without a specific cause of death was regarded as cancer in origin. Death was judged to be due to cardiac events, if it followed acute myocardial infarction, heart failure, or ventricular arrhythmia. Final classifications for the causes of deaths were made on the basis of the full consensus of the independent clinical event committee.

The secondary outcome measures in the current study were recurrent VTE and major bleeding at 90 days after the first recurrent VTE. Recurrent VTE was defined as PE and/or DVT with symptoms accompanied by objective imaging examinations or autopsy^14^. Major bleeding was defined as International Society of Thrombosis and Hemostasis (ISTH) major bleeding, which consisted of a reduction in the hemoglobin level by at least 2 g/dL, transfusion of at least 2 units of blood or symptomatic bleeding in a critical area or organ^16^.

### Statistical analysis

In the current study, we evaluated the baseline characteristics among the 3 groups. Characteristics, management strategies, and outcomes of the first recurrent VTE were compared between the 2 groups of patients who had recurrent VTE during and after discontinuation of anticoagulation therapy. Moreover, we conducted an exploratory analysis for first recurrent VTE during anticoagulation therapy comparing patients with and without active cancer. Categorical variables are presented as numbers and percentages, and continuous variables are presented as the mean and standard deviation or the median and interquartile range (IQR) based on their distributions. Categorical variables were compared using the chi-squared test when appropriate; otherwise, Fisher’s exact test was used. Continuous variables were compared using Wilcoxon’s rank sum test between the 2 groups and one-way analysis of variance or Kruskal–Wallis test based on their distributions among the 3 groups. The 90-day clinical outcomes after the first recurrent VTE are presented as the number of events and incidences with 95% confidence intervals (CI). All statistical analyses were conducted using JMP version 14.0.0 (SAS Institute Inc., Cary, NC, USA; https://www.jmp.com/en_gb/software/new-release/preview-jmp14.html). All reported P-values were 2-tailed, and P-values < 0.05 were considered statistically significant.

## Results

### Patient characteristics

The mean age of the study population was 67 years, 61% were women, and mean body weight and body mass index were 57.9 kg and 23.2 kg/m^2^, respectively. There were 110 patients (3.6%) with first recurrent VTE during anticoagulation therapy, 116 patients (3.8%) with first recurrent VTE after discontinuation of anticoagulation therapy, and 2801 patients (93%) without recurrent VTE during the follow-up period (Fig. 1).

The patient characteristics were different in several aspects across the 3 groups (Table 1). Patients with first recurrent VTE during anticoagulation therapy were younger than patients with first recurrent VTE after discontinuation of anticoagulation therapy, and patients without recurrent VTE during the follow-up period (62.4, 63.9 and 67.5 years, P < 0.001), and more often had active cancer (45, 25 and 22%, P < 0.001) and thrombophilia (12, 5.2 and 4.6%, P = 0.002).

**Table 1:** Patient characteristics.

	First recurrent VTE during anticoagulation therapy (N = 110)	First recurrent VTE after discontinuation of anticoagulation therapy (N = 116)	No recurrent VTE during the follow-up period (N = 2801)	P-value
**Baseline characteristics**				
Age (years)	62.4 ± 16.1	63.9 ± 15.8	67.5 ± 15.3	< 0.001
Women	72 (65%)	72 (62%)	1714 (61%)	0.66
Body weight (kg)	58.6 ± 13.6	58.4 ± 13.3	57.8 ± 13.8	0.79
Body mass index (kg/m^2^)	23.0 ± 3.9	22.8 ± 4.1	23.2 ± 4.5	0.59
**Comorbidities**				
Hypertension	36 (33%)	40 (34%)	1085 (39%)	0.30
Diabetes mellitus	9 (8.2%)	13 (11%)	364 (13%)	0.29
Chronic kidney disease	25 (23%)	36 (31%)	511 (18%)	0.002
Active cancer at diagnosis	49 (45%)	29 (25%)	617 (22%)	< 0.001
Chronic lung disease	4 (3.6%)	15 (13%)	252 (9.0%)	0.048
Heart failure	1 (0.9%)	1 (0.9%)	99 (3.5%)	0.10
History of stroke	11 (10%)	11 (9.5%)	248 (8.9%)	0.90
Connective tissue disease	14 (13%)	11 (9.5%)	219 (7.8%)	0.15
History of major bleeding	8 (7.3%)	13 (11%)	210 (7.5%)	0.33
Unprovoked VTE	35 (32%)	62 (53%)	1345 (48%)	0.002
**Presentation**				
PE with or without DVT	75 (68%)	62 (53%)	1578 (56%)	0.04
DVT only	35 (32%)	54 (47%)	1223 (44%)	
**Laboratory test results at diagnosis**				
Anemia	60 (55%)	59 (51%)	1508 (54%)	0.81
Thrombocytopenia	9 (8.2%)	3 (2.6%)	155 (5.5%)	0.18
D-dimer (μg/mL) (N = 2852)	15.7 (7.8–31.0)	9.0 (4.9–19.3)	10.0 (5.0–20.2)	< 0.001
Thrombophilia	13 (12%)	6 (5.2%)	128 (4.6%)	0.002
**Treatment in the acute phase**				
Initial parenteral anticoagulation therapy	95 (86%)	87 (75%)	2352 (84%)	0.03
Thrombolysis	19 (17%)	23 (20%)	388 (14%)	0.13
Inferior vena cava filter use	37 (34%)	22 (19%)	661 (24%)	0.02
**Anticoagulation therapy beyond the acute phase**	110 (100%)	98 (84%)	2601 (93%)	0.002
Warfarin	96 (87%)	90 (78%)	2490 (89%)	0.01
DOAC	5 (4.6%)	3 (2.6%)	70 (2.5%)	
Heparin	9 (8.3%)	5 (4.3%)	41 (1.5%)	
Presumed stable PT-INR values for warfarin users (N = 2027)	1.8 (1.4–2.2)	1.8 (1.5–2.1)	1.8 (1.5–2.3)	0.57
PT-INR < 1.5 (N = 2027)	23 (31%)	22 (26%)	483 (26%)	0.65
PT-INR < 2.0 (N = 2027)	44 (59%)	56 (67%)	1136 (61%)	0.51

Categorical variables are presented as numbers and percentages, and continuous variables are presented as the mean and standard deviation or the median and interquartile range based on their distributions. Categorical variables were compared using the chi-squared test when appropriate; otherwise, Fisher’s exact test was used. Continuous variables were compared using one-way analysis of variance or Kruskal–Wallis test based on their distributions.

Chronic kidney disease was diagnosed if there was persistent proteinuria or if estimated glomerular filtration rate was < 60 mL/min/1.73 m^2^ for more than 3 months. Patients with active cancer were defined as those on treatment for cancer such as chemotherapy or radiotherapy, those scheduled to undergo cancer-surgery, those with metastasis to other organs, and/or those with terminal cancer (expected life expectancy of 6 months or less) at the time of the diagnosis. Unprovoked VTE was defined as VTE without active cancer nor transient risk factors for VTE. Anemia was defined as hemoglobin level < 13 g/dL for men and < 12 g/dL for women. Thrombocytopenia was defined as platelet count < 100 × 10^9^/L. Thrombophilia included protein C deficiency, protein S deficiency, and antithrombin deficiency. The values of presumed stable INR for warfarin users was evaluated as the first INR between 1 and 6 months after VTE diagnosis, and if there was no available INR between 1 and 6 month after VTE diagnosis, it was regarded as missing.

*VTE* venous thromboembolism, *DVT* deep vein thrombosis, *PE* pulmonary embolism, *DOAC* direct oral anticoagulant, *PT-INR* prothrombin time-international normalized ratio.

### First recurrent VTE events and management strategies

Regarding the timing of first recurrent VTE, 60% (66 patients) of first recurrent VTE during anticoagulation therapy and 19% (22 patients) of first recurrent VTE after discontinuation of anticoagulation therapy occurred within 90 days after the VTE diagnosis (Table 2). Among 110 patients with first recurrent VTE during anticoagulation therapy, 84 patients (76%) received warfarin at first recurrent VTE with the median PT-INR value at recurrent VTE events of 1.6. Regarding the anticoagulation strategies after first recurrent VTE events, 85 patients (77%) continued the same anticoagulant as used at the first recurrent VTE event, whereas 25 patients (23%) switched to a different anticoagulant.

**Table 2:** Characteristics and management strategies of first recurrent VTE events.

	First recurrent VTE during anticoagulation therapy (N = 110)	First recurrent VTE after discontinuation of anticoagulation therapy (N = 116)	P-value
**Timing of first recurrent VTE events**			
Within 90 days after VTE diagnosis	66 (60%)	22 (19%)	< 0.001
Days from anticoagulation discontinuation to first recurrent VTE events	–	120 (30–549)	–
**Anticoagulants at first recurrent VTE events**			
Warfarin	84 (76%)	–	–
PT-INR at recurrent VTE (N = 76)	1.6 (1.3–2.4)	–	–
PT-INR < 1.5 at recurrent VTE (N = 76)	32 (42%)	–	–
PT-INR < 2.0 at recurrent VTE (N = 76)	49 (64%)	–	–
DOAC	5 (4.6%)	–	–
Heparin	21 (19%)	–	–
**Presentation of first recurrent VTE events**			
PE with or without DVT	56 (51%)	52 (45%)	0.36
Hypoxemia	34/56 (61%)	22/52 (42%)	0.06
Shock	18/56 (32%)	7/52 (13%)	0.02
Cardiac arrest/collapse	13/56 (23%)	5/52 (9.6%)	0.07
**Laboratory test results at first recurrent VTE events**			
D-dimer (μg/mL) (N = 169)	10.8 (5.5–30.6)	9.2 (4.3–13.9)	0.02
**Treatment in the acute phase at first recurrent VTE events**			
Initial parenteral anticoagulation therapy	72 (65%)	63 (54%)	0.09
Thrombolysis	16 (15%)	11 (9.5%)	0.24
Inferior vena cava filter use	42 (38%)	14 (12%)	< 0.001
Ventilator support	7 (6.4%)	1 (0.9%)	0.03
Percutaneous cardiopulmonary support	7 (6.4%)	0 (0%)	0.006
**Anticoagulation strategies after first recurrent VTE events**			
Continuation of the same anticoagulant as the anticoagulant at the first recurrent VTE event	85 (77%)	–	–
Warfarin	73/84 (87%)	–	–
DOAC	2/5 (40%)	–	–
Heparin	10/21 (48%)	–	–
Switch from the anticoagulant at the first recurrent VTE event to a different anticoagulant	25 (23%)	–	–
From warfarin to DOAC	2/84 (2.4%)	–	–
From warfarin to heparin	9/84 (11%)	–	–
From DOAC to warfarin	2/5 (40%)	–	–
From DOAC to heparin	1/5 (20%)	–	–
From heparin to warfarin	11/21 (52%)	–	–
From heparin to DOAC	0/21 (0%)	–	–
**All-cause death within 48 h after first recurrent VTE events**	11 (10%)	5 (4.3%)	0.10

Categorical variables are presented as numbers and percentages, and continuous variables are presented as the median and interquartile range. Categorical variables were compared using the chi-squared test when appropriate; otherwise, Fisher’s exact test was used. Continuous variables were compared using Wilcoxon’s rank sum test.

*VTE* venous thromboembolism, *PT-INR* prothrombin time-international normalized ratio, *DOAC* direct oral anticoagulant, *DVT* deep vein thrombosis, *PE* pulmonary embolism.

Among patients with first recurrent VTE during anticoagulation therapy, patients with active cancer had a significantly higher median PT-INR value at recurrent VTE for warfarin users compared with those without active cancer (2.0 versus 1.4, P < 0.001) (Table 3).

**Table 3:** Characteristics and management strategies of first recurrent VTE during anticoagulation therapy comparing patients with and without active cancer.

	Patients with active cancer (N = 49)	Patients without active cancer (N = 61)	P-value
**Timing of first recurrent VTE events**			
Within 90 days after VTE diagnosis	35 (71%)	31 (51%)	0.03
**Anticoagulants at first recurrent VTE events**			
Warfarin	45 (92%)	39 (64%)	0.001
PT-INR at recurrent VTE events (N = 76)	2.0 (1.5–3.0)	1.4 (1.3–1.9)	< 0.001
PT-INR < 1.5 at recurrent VTE events (N = 76)	10 (24%)	22 (65%)	< 0.001
PT-INR < 2.0 at recurrent VTE events (N = 76)	21 (50%)	28 (82%)	0.004
DOAC	2 (4.1%)	3 (4.9%)	0.83
Heparin	2 (4.1%)	19 (31%)	< 0.001
**Anticoagulation strategies after first recurrent VTE events**			
Continuation of the same anticoagulant as the anticoagulant at the first recurrent VTE event	39 (80%)	46 (75%)	0.60
Warfarin	37/45 (82%)	36/39 (92%)	–
DOAC	1/2 (50%)	1/3 (33%)	
Heparin	1/2 (50%)	9/19 (47%)	
Switch from the anticoagulant at the first recurrent VTE event to a different anticoagulant	10 (20%)	15 (25%)	0.60
From warfarin to DOAC	0/45 (0%)	2/39 (5.1%)	–
From warfarin to heparin	8/45 (18%)	1/39 (2.6%)	
From DOAC to warfarin	1/2 (50%)	1/3 (33%)	
From DOAC to heparin	0/2 (0%)	1/3 (33%)	
From heparin to warfarin	1/2 (50%)	10/19 (53%)	
From heparin to DOAC	0/2 (0%)	0/19 (0%)	
**All-cause death within 48 h after first recurrent VTE events**	4 (8.2%)	7 (11.5%)	0.75

Categorical variables are presented as numbers and percentages, and continuous variables are presented as the median and interquartile range. Categorical variables were compared using the chi-squared test when appropriate; otherwise, Fisher’s exact test was used. Continuous variables were compared using Wilcoxon’s rank sum test.

*VTE* venous thromboembolism, *PT-INR* prothrombin time-international normalized ratio, *DOAC* direct oral anticoagulant.

### Clinical outcomes after first recurrent VTE events

Among the patients with recurrent VTE, 23 patients (20.9%) during anticoagulation therapy, and 24 patients (20.7%) after discontinuation of anticoagulation therapy died within 90 days after the first recurrent VTE events (Table 4). Of the 23 deaths after first recurrent VTE during anticoagulation therapy, deaths due to PE (fatal PE) accounted for 13 (57%), and deaths due to cancer accounted for 9 (39%). Of the 24 deaths after first recurrent VTE after discontinuation of anticoagulation therapy, deaths due to PE (fatal PE) accounted for 6 (25%), and deaths due to cancer accounted for 11 (46%). Patients with first recurrent VTE during anticoagulation therapy experienced recurrent VTE in 1 patients (0.9%) and major bleeding in 9 patients (8.2%) at 90 days after the first recurrent VTE events (Table 4). Patients with first recurrent VTE after discontinuation of anticoagulation therapy experienced recurrent VTE in 1 patients (0.9%) and major bleeding in 8 patients (6.9%) at 90 days after the first recurrent VTE events.

**Table 4:** Clinical outcomes at 90 days after first recurrent VTE events.

	First recurrent VTE during anticoagulation therapy (N = 110)	First recurrent VTE after discontinuation of anticoagulation therapy (N = 116)
**All-cause death**	23 (20.9 [14.3–29.5]%)	24 (20.7 [14.3–29.0]%)
**Causes of death**		
Fatal PE	13/23 (57%)	6/24 (25%)
Bleeding events	1/23 (4.4%)	3/24 (13%)
Cancer	9/23 (39%)	11/24 (46%)
Cardiac events	0/23 (0%)	0/24 (0%)
Other non-cardiac events	0/23 (0%)	3/24 (13%)
Unknown	0/23 (0%)	1/24 (4.2%)
**Recurrent VTE**	1 (0.9 [0.0–5.5]%)	1 (0.9 [0.0–5.2]%)
**Major bleeding**	9 (8.2 [4.2–15.0]%)	8 (6.9 [3.3–13.2]%)

The 90-day clinical outcomes after the first recurrent VTE are presented as the number of events and incidences with 95% confidence intervals.

Death was judged to be due to PE (fatal PE), if it was confirmed by autopsy or if death followed a clinically severe PE after first recurrent PE events. Death was judged to be bleeding-related, if it followed an intracranial haemorrhage or a bleeding episode leading to hemodynamic deterioration. Death from end-stage cancer without a specific cause of death was regarded as of cancer origin. Death was judged to be due to cardiac events, if it followed acute myocardial infarction, heart failure, or ventricular arrhythmia.

*VTE* venous thromboembolism, *PE* pulmonary embolism.

Among the patients with first recurrent VTE during anticoagulation therapy, 15 patients (30.6%) with active cancer, and 8 patients (13.1%) without active cancer died within 90 days after the first recurrent VTE events (Table 5). Of the 15 deaths among patients with active cancer, deaths due to PE (fatal PE) accounted for 5 (33%) and deaths due to cancer accounted for 9 (60%), while all 8 deaths among patients without active cancer were due to PE (fatal PE).

**Table 5:** Clinical outcomes at 90 days after first recurrent VTE events during anticoagulation therapy comparing patients with and without active cancer.

	Patients with active cancer (N = 49)	Patients without active cancer (N = 61)
**All-cause death**	15 (30.6 [19.4–44.6]%)	8 (13.1 [6.5–24.1]%)
**Causes of death**		
Fatal PE	5/15 (33%)	8/8 (100%)
Bleeding events	1/15 (6.7%)	0/8 (0%)
Cancer	9/15 (60%)	0/8 (0%)
Cardiac events	0/15 (0%)	0/8 (0%)
Other non-cardiac events	0/15 (0%)	0/8 (0%)
Unknown	0/15 (0%)	0/8 (0%)
**Recurrent VTE**	0 (0.0 [0.0–8.7]%)	1 (1.6 [0.0–9.6]%)
**Major bleeding**	5 (10.2 [4.0–22.2]%)	4 (6.6 [2.1–16.1]%)

The 90-day clinical outcomes after the first recurrent VTE are presented as the number of events and incidences with 95% confidence intervals.

Death was judged to be due to PE (fatal PE), if it was confirmed by autopsy or if death followed a clinically severe PE after first recurrent PE events. Death was judged to be bleeding-related, if it followed an intracranial haemorrhage or a bleeding episode leading to hemodynamic deterioration. Death from end-stage cancer without a specific cause of death was regarded as of cancer origin. Death was judged to be due to cardiac events, if it followed acute myocardial infarction, heart failure, or ventricular arrhythmia.

*VTE* venous thromboembolism, *PE* pulmonary embolism.

## Discussion

The main findings of the current study were as follows; (1) Patients with first recurrent VTE during anticoagulation therapy had a significantly higher prevalence of active cancer compared with those after discontinuation of anticoagulation therapy; (2) The PT-INR value for warfarin users at recurrent VTE events in patients during anticoagulation therapy was significantly higher in patients with active cancer than in patients without active cancer; (3) Approximately 20% of patients died within 90 days after the first recurrent VTE events, and fatal PE and cancer were the major causes of deaths.

Generally, recurrent VTE after discontinuation of anticoagulation therapy might be managed by restart of anticoagulation therapy with indefinite duration^7–10^. The clinical dilemma is the optimal anticoagulation strategies for recurrent VTE during anticoagulation therapy^6^. Recurrent VTE in the acute phase soon after initiation of anticoagulation therapy seems to be managed by more aggressive antithrombotic therapy including thrombolysis, increased intensity of anticoagulation therapy, and switching from oral anticoagulants to parenteral anticoagulants. However, currently, there have been no randomized clinical trials that have evaluated management strategies for patients with recurrent VTE during anticoagulant therapy. Potential causes of recurrent VTE during anticoagulation therapy could be divided into patient-related factors and treatment-related factors^6^. The investigation of the potential causes could be important to consider the appropriate management strategies after recurrent VTE during anticoagulation therapy.

Several patient-related factors including cancer, thrombophilia, autoimmune disease, pregnancy, and vascular abnormalities were reported as the causes of recurrent VTE^17–21^. Of these, the most important patient-related factor is thought to be active cancer^9^. A previous study reported that 31% of patients with recurrent VTE during anticoagulation therapy had active cancers^17^. Consistent with the previous report, the current study showed a high prevalence (45%) of patients with recurrent VTE during anticoagulation therapy. Patients with active cancer were reported to receive sub-optimal intensity of anticoagulation therapy for warfarin users due to high-bleeding risk and drug interactions of anti-cancer drug^22^. However, the current study showed that warfarin users with active cancer had higher PT-INR values compared with those without, suggesting the importance of active cancer as the patient-related factor of recurrent VTE on appropriate anticoagulation therapy. There might be no established anticoagulation strategies for patients with recurrent VTE during appropriate anticoagulation therapy. A current guideline weakly recommends switching from oral anticoagulants to low molecular weight heparin (LMWH) at least temporarily and increasing the dose of LMWH for LMWH users based on a low level of evidence^9^, although there are no recommendations for long-term anticoagulation strategies. The current study showed that a majority of patients with recurrent VTE during anticoagulation therapy were treated with parenteral anticoagulation therapy or thrombolysis in the acute phase, and continued the same anticoagulant as used at the recurrent VTE event beyond the acute phase.

Treatment-related factors could include sub-therapeutic intensity of warfarin, inappropriate reduced dosage of direct oral anticoagulants (DOAC), concomitant drugs that reduce anticoagulant effect, and patient adherence to drug^9^. The recommended target PT-INR for VTE in the Japanese guidelines is 2.0 (range, 1.5–2.5) without established evidences^7^, which is lower than the target value (PT-INR, 2.5; range, 2.0–3.0) used in other Western countries^8–10^. The current study showed that there was no significant difference in the median presumed stable PT-INR values for warfarin users among the 3 groups (1.8, 1.8 and 1.8, P = 0.57). However, the current study also showed that the median PT-INR value at recurrent VTE events for warfarin users was 1.6 and more than half of patients had PT-INR values less than 2.0 at recurrent VTE events, and low PT-INR value at recurrent VTE events was more remarkable in patients without active cancer. These results could suggest that sub-therapeutic intensity of warfarin might be one of the causes of recurrent VTE during anticoagulation therapy as treatment-related factor especially in patients without active cancer. Clinicians should be notified for the importance of maintenance of appropriate intensity of anticoagulation therapy during the follow-up. These issues should be confirmed by further studies in the future.

The clinical outcomes after recurrent VTE could also be important in considering the appropriate management strategies after recurrent VTE. A previous study reported that patients with recurrent VTE on vitamin K antagonists (warfarin) therapy have a worse prognosis with an increased risk of both further recurrences (6.8%) and major bleeding (2.3%) at 3 months compared with patients with recurrent VTE during vitamin K antagonists therapy^17^. Another study reported that a considerably high risk of recurrence (8.6%) and major bleeding (1.4%) during 3 months in 70 cancer patients with recurrent VTE during anticoagulation therapy who either switched from warfarin to LMWH or had their LMWH dose increased^23^. The current study showed a relatively low risk of further recurrence (0.9%) and high risk of major bleeding (8.2%) at 90 days in patients with recurrent VTE during anticoagulation therapy. There results could be partly due to a relatively frequent usage of thrombolysis in the acute phase (15%) and different ethnicity between Whites and Asians^24^. The current study also showed that a considerably high proportion of patients (20.9%) died within 90 days after the recurrent VTE during anticoagulation therapy and fatal PE and cancer were major causes of deaths. A major cause of death was cancer among patients with active cancer, while all deaths among patients without active cancer was fatal PE. Clinicians should be careful for avoiding inappropriate anticoagulation strategies to prevent recurrent fatal PE.

### Study limitations

The current study has several limitations. First, the current study was an observational study, which can be subject to various biases inherent to observational study design. The therapeutic decision-making was left to the discretion of the attending physicians, which could have influences on clinical outcomes after recurrent VTE events. Second, detailed management strategies after recurrent VTE such as dosing of heparin and warfarin were not evaluated in the current study. Third, demographics, practice patterns as well as clinical outcomes in patients with VTE in Japan may be different from those outside Japan. Finally, the current study was conducted before introduction of DOACs for VTE in Japan. Thus, it should be interpreted with caution whether the present results could be extrapolated to patients treated with DOACs.

## Conclusions

In this real-world large registry, active cancer was a major cause of recurrent VTE during anticoagulation therapy as a patient-related factor, while sub-optimal intensity of anticoagulation therapy was a major cause of recurrent VTE during anticoagulation therapy as a treatment-related factor, particularly in patients without active cancer.

## Supplementary Information

Supplementary Information.

## Data Availability

If the relevant review board or ethics committee approve the data sharing and all investigators of the COMMAND VTE Registry give their consent, the deidentified participant data will be shared on a request basis through the principal investigator (Yugo Yamashita: yyamashi@kuhp.kyoto-u.ac.jp). Study protocol and statistical analysis plan will also be available. The data will be shared as Excel files via E-mail during the proposed investigation period.
