# Supplementary material for: Clinical characteristics, management strategies and outcomes of patients with recurrent venous thromboembolism in the real world
Source: Sci Rep. 2022 Dec 23;12:22437. doi: 10.1038/s41598-022-26947-9 (PMC9794688; doi:10.1038/s41598-022-26947-9)
Supplement: Supplementary file 1 — Supplementary Information. [file 41598_2022_26947_MOESM1_ESM.pdf]

Supplementary File

Supplementary Appendix 1: List of participating centers and investigators

Graduate School of Medicine, Kyoto University: Yugo Yamashita, Shintaro Yamagami, Takanori Aizawa, Tomohiko Taniguchi, Hiroki Shiomi, Takao Kato, Naritatsu Saito, Takeru Makiyama, Satoshi Shizuta, Koh Ono, Takeshi Kimura (Kyoto University Hospital Ethics Committee; the approval number R0493)

Kurashiki Central Hospital: Hidewo Amano, Reo Hata, Kazuki Matsushita, Osakada Kohei, Sano Arata, Kazushige Kadota (Kurashiki Central Hospital Ethics Committee; the approval number 2179)

Kinki University Hospital: Toru Takase, Tomoyuki Ikeda, Syunichi Miyazaki (Kinki University Ethics Committee; the approval number 28-056)

Kokura Memorial Hospital: Seiichi Hiramori, Kenji Ando (Kokura Memorial Hospital Ethics Committee; the approval number 16051804)

Kobe City Medical Center General Hospital: Kitae Kim, Yutaka Furukawa (Kobe City Medical Center General Hospital Ethics Committee; the approval number zn160801)

Japanese Red Cross Otsu Hospital: Ritsuya Tachiiri, Takashi Konishi (Japanese Red Cross Otsu Hospital Ethics Committee; the approval number 373)

National Hospital Organization Kyoto Medical Center: Yuji Tezuka, Masaharu Akao (Kyoto Medical Center Ethics Committee; the approval number 16-021)

Osaka Red Cross Hospital: Yohei Kobayashi, Tsukasa Inada (Osaka Red Cross Hospital Ethics Committee; the approval number 699)

Shiga Medical Center for Adults: Takeshi Inoue, Yukio Noma, Shigeru Ikeguchi (Shiga Medical Center Ethics Committee; the approval number 20160603:154: Contemporary Management And Outcomes In Patients With Venous Thromboembolism Registry)

Japanese Red Cross Wakayama Medical Center: Maki Oi, Yasuyo Motohashi, Mamoru Toyofuku, Takashi Tamura (Japanese Red Cross Wakayama Medical Center Ethics Committee; the approval number 414)

The Tazuke Kofukai Medical Research Institute, Kitano Hospital: Toshiaki Izumi, Nozomi Tanaka, Moriaki

inoko Kitano Hospital Ethics Committee; the approval number P16-05-16)

Shizuoka General Hospital: Tomoya Tada, Kotaro Takahashi, Hiroki Sakamoto (Shizuoka General Hospital Ethics Committee; the approval number SGHIRB#2016011)

Osaka Saiseikai Noe Hospital: Po-Min Chen, Ichiro Kouchi (Osaka Saiseikai Noe Hospital Ethics Committee; the approval number 28-3)

Shizuoka City Shizuoka Hospital: Koichiro Murata, Tomoya Onodera (Shizuoka City Shizuoka Hospital Ethics Committee; the approval number 1606)

Shimada Municipal Hospital: Yoshiaki Tsuyuki, Takeshi Aoyama (Shimada Municipal Hospital Ethics Committee; the approval number 28-1)

Shiga University of Medical Science: Hiroshi Sakai, Minoru Horie (Shiga University Ethics Committee; the approval number 28-078)

Hyogo Prefectural Amagasaki General Medical Center: Syunsuke Saga, Yukihito Sato (Hyogo Prefectural Amagasaki General Medical Center Ethics Committee; the approval number 28-9)

Kishiwada City Hospital: Tomoki Sasa, Mitsuo Matsuda (Kishiwada City Hospital Ethics Committee; the approval number H28-4-25 Kishibyourin No.1: Contemporary Management And Outcomes In Patients With Venous Thromboembolism Registry)

Tenri Hospital: Jiro Sakamoto, Yoshihisa Nakagawa (Tenri Hospital Ethics Committee; the approval number 753)

Kyoto Okamoto Memorial Hospital: Chinatsu Yamada, Osamu Doi (Kyoto Okamoto Memorial Hospital Ethics Committee; the approval number 2016-07)

Nishikobe Medical Center: Minako Kinoshita, Yuko Yoshigai, Keiichiro Yamane, Naoki Yoshino, Mitsunori Kawato, Junya Ejiri, Hiroshi Eizawa (Nishikobe Medical Center Ethics Committee; the approval number 2016-04)

Nara Hospital, Kinki University Faculty of Medicine: Kiyonori Togi, Manabu Shirotani (Nara Hospital, Kinki University Faculty of Medicine Ethics Committee; the approval number 370)

Hikone Municipal Hospital: Tomoyuki Ikeda, Yagi Mitsuyo, Nakatani Misaki, Yoshihiro Himura (Hikone

Municipal Hospital Ethics Committee; the approval number 28-6)

Kansai Electric Power Hospital: Katsuhisa Ishii (Kansai Electric Power Hospital Ethics Committee; the approval number 28-13)

Mitsubishi Kyoto Hospital: Kazuhisa Kaneda, Shinji Miki (Mitsubishi Kyoto Hospital Ethics Committee; the approval number 16-3)

Koto Memorial Hospital: Hiroshi Mabuchi, Tomoyuki Murakami (Koto Memorial Hospital Ethics Committee; the approval number 2016: Contemporary Management And Outcomes In Patients With Venous Thromboembolism Registry)

Sugita Genpaku Memorial Obama Municipal Hospital: Hideo Otani (Obama Municipal Hospital Ethics Committee; the approval number 28-2)

Hirakata Kohsai Hospital: Kensuke Takabayashi, Shoji Kitaguchi (Hirakata Kohsai Hospital Ethics Committee; the approval number H28-5-19: Contemporary Management And Outcomes In Patients With Venous Thromboembolism Registry)

Shimabara Hospital: Yoshiki Matoba, Mamoru Takahashi (Kyoto University Hospital Ethics Committee; the approval number R0493-1)

Supplementary Appendix 2: Definitions for patient characteristics

Hypertension was diagnosed if peripheral blood pressure was >140/90 mmHg or if the patient was taking medication for hypertension. The presence of diabetes was diagnosed using hemoglobin A1c (HbA1c) [National Glycohemoglobin Standardization Program (NGSP), 6.5%] as the standard or was assumed if the patient was taking medication for the treatment of diabetes. Chronic kidney disease was diagnosed if there was persistent proteinuria or if estimated glomerular filtration rate (eGFR) was <60 mL/min/1.73 m^2 for more than 3 months. The values of eGFR were calculated based on the equation reported by Japan Association of Chronic Kidney Disease Initiative [male: $194*Scr^{-1.094}*age^{-0.287}$, female: $194*Scr^{-1.094}*age^{-0.287}*0.739$]. Chronic lung disease was defined as persistent lung disorders such as asthma, chronic obstructive pulmonary disease, and restrictive lung diseases. Heart failure was diagnosed if the patient had a history of hospitalization for heart failure, if the patient had symptoms due to heart failure [New York Heart Association (NYHA) functional class ≥2], or if the left ventricular ejection fraction was <40%. History of major bleeding was diagnosed if the patient had a history of International Society of Thrombosis and Hemostasis (ISTH) major bleeding, which consisted of a reduction in the hemoglobin level by at least 2 g/dL, transfusion of at least 2 units of blood or symptomatic bleeding in a critical area or organ[1]. Transient risk factors for venous thromboembolism (VTE) included recent surgery (within 2 months prior to VTE), recent immobilization (defined as non-surgical bed-ridden patients with bathroom privileges for >4 days within 2 months prior to VTE), long-distance travel (travel lasting >=6 hours in the previous 3 weeks), central venous catheter use, pregnancy or puerperium, recent leg trauma, fracture or burn (any events requiring immobilization in the past 2 months), severe infection, and estrogen use[2]. Unprovoked VTE was defined as VTE without active cancer nor transient risk factors for VTE. Proximal deep vein thrombosis (DVT) was defined as venous thrombosis which was located in popliteal, femoral, or iliac veins. Anemia was defined as hemoglobin level <13 g/dL for men and <12 g/dL for women. Thrombocytopenia was defined as platelet count $<100\times10^9$/L. Thrombophilia included protein C deficiency, protein S deficiency, and antithrombin deficiency.

Supplementary Appendix 3: The independent clinical event committee

Hidenori Yaku, MD (Graduate School of Medicine, Kyoto University), Yusuke Yoshikawa, MD (Graduate School of Medicine, Kyoto University), Kosuke Doi, MD (National Hospital Organization Kyoto Medical Center), Kensuke Takabayashi, MD (Hirakata Kohsai Hospital)

Supplementary Reference

1. Schulman S, Kearon C. Definition of major bleeding in clinical investigations of antihemostatic medicinal products in non-surgical patients. J Thromb Haemost 2005;3:692-694.

2. Laporte S, Mismetti P, Decousus H, et al. Clinical predictors for fatal pulmonary embolism in 15,520 patients with venous thromboembolism: findings from the Registro Informatizado de la Enfermedad TromboEmbolica venosa (RIETE) Registry. Circulation 2008;117:1711-1716.
